# Modulation of HERV Expression by Four Different Encephalitic Arboviruses during Infection of Human Primary Astrocytes

**DOI:** 10.3390/v14112505

**Published:** 2022-11-12

**Authors:** Fernando Luz de Castro, Otávio José Bernandes Brustolini, Victor Emmanuel Viana Geddes, Jorge Paes Barreto Marcondes de Souza, Soniza Vieira Alves-Leon, Renato Santana Aguiar, Ana Tereza Ribeiro Vasconcelos

**Affiliations:** 1 D’Or Institute for Research and Education (IDOR), Rio de Janeiro 22281-100, Brazil; 2 Bioinformatics Lab, National Laboratory for Scientific Computing (LNCC), Petrópolis 25651-076, Brazil; 3 Integrative Biology Lab, Department of Genetics, Ecology and Evolution, Institute of Biological Sciences, Federal University of Minas Gerais (UFMG), Belo Horizonte 31270-901, Brazil; 4 Service of Neurosurgery, Department of Surgery, University Hospital Clementino Fraga Filho, Federal University of Rio de Janeiro (UFRJ), Rio de Janeiro 21941-617, Brazil; 5 Laboratory of Translational Neurosciences (LABNET/UNIRIO), Federal University of the State of Rio de Janeiro, Rio de Janeiro 20270-004, Brazil

**Keywords:** human endogenous retroviruses, arboviruses, astrocytes

## Abstract

Human retroelements (HERVs) are retroviral origin sequences fixed in the human genome. HERVs induction is associated with neurogenesis, cellular development, immune activation, and neurological disorders. Arboviruses are often associated with the development of encephalitis. The interplay between these viruses and HERVs has not been fully elucidated. In this work, we analyzed RNAseq data derived from infected human primary astrocytes by Zika (ZikV), Mayaro (MayV), Oropouche (OroV) and Chikungunya (ChikV) viruses, and evaluated the modulation of HERVs and their nearby genes. Our data show common HERVs expression modulation by both alphaviruses, suggesting conserved evolutionary routes of transcription regulation. A total of 15 HERVs were co-modulated by the four arboviruses, including the highly upregulated HERV4_4q22. Data on the upregulation of genes nearby to these elements in ChikV, MayV and OroV infections were also obtained, and interaction networks were built. The upregulation of 14 genes common among all viruses was observed in the networks, and 93 genes between MayV and ChikV. These genes are related to cellular processes such as cellular replication, cytoskeleton, cell vesicle traffic and antiviral response. Together, our results support the role of HERVs induction in the transcription regulation process of genes during arboviral infections.

## 1. Introduction

During the evolutionary history of species, retroviruses have been integrated into the host genomes, driving genes and chromosome evolution. After integration, retroviruses suffer a series of genetic alterations that limit the cell’s ability to complete the exogenous cycle, in a process called retrovirus endogenization. These agents comprise the group of endogenous retroviruses, or ERVs, which can be found at different levels in virtually all vertebrates and even in some invertebrate species [1]. Once integrated, ERVs are retrotransposable elements that can potentially modulate the expression of neighboring genes (proximal and distal), bringing new promoters and epigenetic markers. In addition, ERV integration also modifies gene expression by acting as coding and non-coding sequences, generating long non-coding RNAs (lncRNAs), endogenous-siRNAs (endo-siRNAs) and PIWI-Interacting RNAs (piRNAs) associated with chromatin topology [2,3,4,5,6,7].

HERVs are human endogenous retroviruses acquired and fixed in the human genome. HERVs are classified as type I class retrotransposons or retrotransposons containing Long Terminal Repeats (LTRs). These sequences make up about 8% of the human genome, and many of them rely only on the presence of solitary LTRs modulating global gene expression [8,9]. The endogenization of HERVs is associated with gain of function, as exemplified by HERV-W and HERV-FDR, which are involved in the formation of the placenta in humans through the expression of its fusogenic proteins (Syncytin-1 and 2, respectively) during pregnancy [10,11,12]. However, HERVs modulation is also associated with pathogenesis, including neurological and psychiatric conditions such as multiple sclerosis (MS), schizophrenia and Alzheimer’s disease (AD) in humans [13]. Antony et al. (2004) reported an increase in the levels of expression of syncytin-1 in glial cells of patients with demyelinating MS, confirmed by functional experiments in mouse models, showing the ability of this HERV protein to activate the nitric oxide synthase promoting inflammatory conditions in the brain [14].

Viruses are the most common infectious agents associated with the development of encephalitis, especially herpesviruses and arthropod-borne viruses (arboviruses). This last group comprises at least 150 members related to human disease, including the genera *Alphavirus*, *Orthobunyavirus* and *Flavivirus* [15,16,17]. Arboviral infections represent a huge public health problem worldwide, and pose a health threat, especially in tropical and equatorial humid countries, such as Brazil [18]. Zika virus (ZikV) and Chikungunya virus (ChikV) were recently introduced in Brazil and are associated with increasing reports of demyelinating and neurological manifestations [19]. During outbreaks of febrile disease, it was also possible to find other agents from different virus families that have been emerging and re-emerging, leading to epidemics in the Amazon region, such as Oropouche virus (OroV) and Mayaro virus (MayV), also associated to brain encephalitis [20]. In addition to classic viral febrile illness symptoms (known as “FAR”, from fever/arthralgia/rash), these viruses could spread to the central nervous system (CNS), leading to several inflammatory-associated neurological conditions [21].

Brain cells, including astrocytes, microglia and oligodendrocytes, play a role in brain homeostasis and immune inflammation. Astrocytes and microglia cells actively participate in the innate immune response triggered via a series of receptors involved in the detection of pathogen-associated molecular patterns (PAMPs), promoting the expression of several interferon-induced genes (ISGs) [22]. Astrocytes are also responsible for maintaining homeostasis in the CNS, providing support for neurons and contributing to the flow and processing of information in the brain [23]. They are located juxtaposed with the endothelial cells of the blood–brain barrier and are susceptible to arbovirus infection [24]. However, the molecular pathways involved in the astrocyte response to virus infection are still under investigation. Most of the studies were performed in murine models and human post-mortem tissues due to the difficulty of obtaining human primary astrocyte cells.

Here, we investigate the HERVs’ modulation by four different encephalitis-associated viruses (ChikV, MayV, OroV and ZikV), and their roles in the gene transcription of nearby genes directly on human astrocytes cells derived from brain surgery. A general picture of the upregulation of HERVs by the four agents was observed, with 15 HERVs being shared among the four viruses, including the highly regulated HERV4_4q22.1. It was also possible to visualize a specific signature in terms of the viral family since both the HERVs and their nearby genes regulated by ChikV and MayV (both alphaviruses) were much more similar to each other than between OroV and ZikV. In summary, the results of this article suggest a possible role of HERVs as important agents in the regulation of gene expression, both during the viral replication cycle as well as in the inflammatory processes triggered in the development of encephalitis caused by these viruses.

## 2. Materials and Methods

### 2.1. Cells Infection and RNAseq

Adult primary human astrocytes were isolated from the normal subcortical tissue of temporal lobectomy done as the selected surgical treatment for patients with drug-refractory epilepsy. All patients gave written consent to the study, and the procedures agreed with the Brazilian Ministry of Health Ethics Committee (Certification of Presentation for Ethical Approval, CAAE, submission number 69409617.9.0000.5258, decision number 2.792.114).

Human primary astrocytes cells were isolated in DMEM/F-12 (Gibco, Thermo Fisher Scientific, Waltham, MA, USA) growth medium supplemented with 10% Fetal Calf Serum (FCS) (Gibco, Thermo Fisher Scientific) in a humidified 5% CO_2_, 95% air atmosphere at 37 °C for 2 h. Human astrocytes from up to the seventh passage and from two different donors were used in the study. The infection by the four arboviruses (ChikV, MayV, OroV and ZikV) occurred in the same way as in the previous experiment [24]. Briefly, astrocyte infections were performed at a multiplicity of infection (MOI) 1 (2 × 105 cells/well) for all four viruses in a 12-well plate over 1 h at 37 °C and 5% CO2 in a medium without FBS. Cells were collected for analysis according to the different hours post-infection (hpi) specific for each virus, as described before [24]. Viral infections were then confirmed by Flow Cytometry and virus infectivity by immunofluorescence using antibodies against each viral protein. RNA was isolated using the RNeasy Mini kit (Qiagen, Hilden, Germany) and quantified by the Qubit RNA HS Assay kit and Qubit 3 Fluorometer (Thermo Fisher Scientific). Ribosomal RNA was depleted using Ribo-Zero Gold (Illumina, San Diego, CA, USA) and 200 ng of total RNA for each sample was used for library preparation with TruSeq Stranded Total RNA Library Prep (Illumina) according to the manufacturer’s instructions. RNA-seq were performed in 2 × 9 samples using NextSeq 500/550 High Output v2 Kit (150 cycles) (Illumina) in a NextSeq 550 platform (Illumina).

### 2.2. Differential HERVs Expression Estimation

The RNA-seq dataset of human primary astrocyte cells effectively infected with arboviruses ChikV, MayV, OroV, and ZikV was previously obtained [24], and was retrieved from the SRA accession PRJNA662366. The reads were submitted to Trimmomatic [25] to trim the adapters and reads with low base quality. The output reads were aligned to the human reference genome hg38 using bowtie2 [26] with the options for sensitive local alignment search (--very-sensitive-local) and up to 100 alignments reported for each fragment pair (-k 100). By applying these aligned map files (BAM) and the HERVs hg38 GTF file (https://github.com/mlbendall/telescope_annotation_db, accessed on 22 June 2022) to the Telescope [27] with the default parameters, we have obtained the HERVs expression table. The HERVs, which have expression values below 0.5 counts per million (CPM), were removed. We applied the R/Bioconductor DESeq2 [28] to calculate the statistical significance of the differential expression analysis. The HERVs were admitted as differentially expressed (DEH) with adjusted *p*-values < 0.1 and |log2 (fold-change)| > 1. The DEH data were stored in a relational database PostgreSQL. An HTML interface allows easy access to the gene’s enrichment with Gene Ontology (GO), KEGG/Pathway, and Reactome. It can be accessed at https://biotools.labinfo.lncc.br/hervs_astrocytesvirus/ (accessed on 14 September 2022).

### 2.3. Correlation between DEHERVs and Nearby DEGs

The differentially expressed genes (DEG) of the ChikV, MayV, OroV, and ZikV were obtained from [24] at the web address http://biotools.labinfo.lncc.br/astrocitovirus_data, accessed on 14 September 2022. These DEGs were correlated to DEHs by comparing their relative loci positions in the chromosomes [24]. Using the two GTF files, one of the human genome hg38 and the other of the annotated HERVs, we recovered the loci positions and calculated the 5′ (upstream) and 3′ (downstream) distances. The upstream distance was measured by a number of nucleotides that separate the stop codon of the gene from the initial nucleotide of the HERV. In the same way, we calculated the downstream distance by counting the number of nucleotides separating the gene’s initial codon from the last nucleotide in the 3′LTR HERV position. Again, all the results of this analysis can be found on the website mentioned before.

### 2.4. The Construction of Protein-Protein Network of the HERVs Nearby Genes

The interaction network of DEHs near to DEGs was created using the protein–protein interactions (PPI) information provided by the Biogrid database (https://thebiogrid.org, accessed on 18 July 2022) version 4.3.194. All the non-expressed DEGs in ChikV, MayV, OroV, and ZikaV were removed. We created an undirected and unweighted network with the expressed genes based on the PPI interactions with the software Cytoscape 3.8.0 [29]. The nodes (genes) were labeled with the gene’s name, the log2 Fold Change, and two centralities: betweenness and eigenvector. The node size is proportional to the expression in |log2FC|. The interaction (edge) is a simple binary relation. The topology was chosen as a BioLayout option with the best visualization.

## 3. Results

### 3.1. Modulation of HERVs Transcription during Arboviral Infections in Astrocytes

To explore arboviral infection’s interference in the HERVs expression in neurologic models, we infected human primary astrocytes with ChikV, MayV (both alphaviruses), OroV (orthobunyavirus), and ZikV (flavivirus). It is important to emphasize that the surgical methodology for obtaining the study cells still allows for the isolation and cultivation of other fundamental cells involved in the inflammatory process in the central nervous system, such as microglia. A recent work evaluating the inflammatory trigger resulting from neuronal infection by OroV in Adult Human Brain Slices found, for example, these cells as protagonists, and did not even find infected astrocytes in these samples specifically [30]. Thus, we propose to extend the approach used in this work for future exploratory studies on the role of these cells in the inflammation triggered by arboviruses.

The differentially expressed retrotranscriptome of infected cells compared to non-infected controls is available in our in-house user-friendly interface, which allows for the entry of keywords, HERVs IDs, or genes nearby. As a result, the expressed values with statistical significance will be returned. This platform even permits a comparison of the nearby genes of the differentially expressed HERVs among the viruses via a Venn diagram and the enrichment of the nearby genes via Gene Ontology (GO), pathways (KEGG), and Reactome (http://biotools.labinfo.lncc.br/hervs_astrocitovirus, accessed on 14 September 2022).

**Figure 1:**
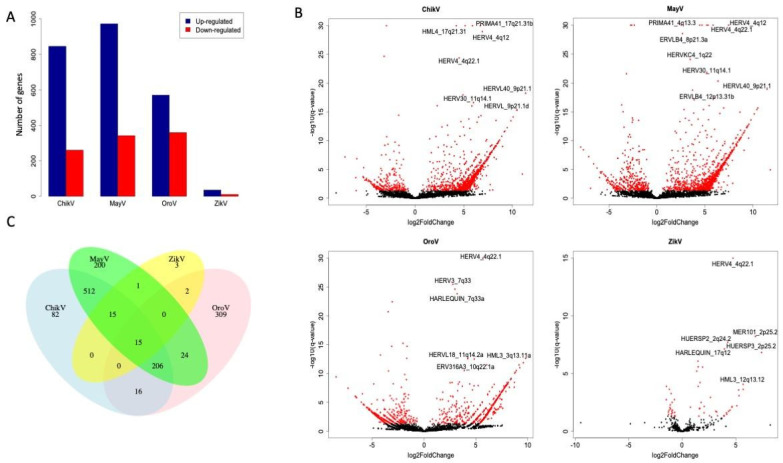
Modulation of HERVs’ expression in human primary astrocytes infected with ChikV, MayV, OroV, and ZikV. (**A**) The number of differentially expressed HERVs comparing the induced (upregulated) and suppressed (downregulated) HERVs by the four viruses. (**B**) Dispersion of the HERVs comparing the log2FC and significance (−log10(q-value)). The red dots indicate the accepted differentially expressed HERVs up to log2FC > 1 and adjusted *p*-value < 0.05. (**C**) Specific co-regulation of HERVs by the four viruses ChikV, MayV, OroV, and ZikV.

We observed a common pattern of higher numbers of HERVs from different families upregulated by the four investigated viruses (Figure 1A,B, Table S1). Even during flavivirus infection (ZikV), which has lower replication rates, we observed a higher proportion of upregulated HERVs, confirming that overall, the viral infections induce HERVs expression. For this reason and considering the importance of HERVs’ upregulation in cellular gene expression, we focused only on the upregulated HERVs. To guarantee the same levels of HERVs’ modulation by viruses, we only analyzed data experiments that reached 80% of infected cells at the same virus infectivity level (MOI 1). However, as previously discussed, flaviviruses such as ZikV present a slow replication rate compared to alphaviruses such as ChikV and MayV, being less productive in astrocyte cells and taking more time to reach the same levels of replication [24]. This may explain, for example, the lower number of HERVs whose expression was detected as significantly modulated by ZikV at the time of analysis (48 hpi). The modulated levels of HERVs agree with the replication rates of each virus used here. As mentioned before, alphavirus infections were more productive in primary astrocytes cells, followed by orthobunyavirus and flavivirus [24].

When we look at the HERVs modulated by each of the four infections, it is interesting to highlight that a total of 15 HERVs were upregulated by the four arboviruses, suggesting a conserved evolutionary route of HERV modulation by them (Table 1). Among these HERVs, HERV4_4q22.1 stands out, as it was highly regulated at very similar levels among the four viruses (Figure 1B,C).

It is also possible to observe a conserved pattern of HERVs’ induction associated with the virus family and, consequently, the genomic replication strategy. MayV and ChikV, both alphaviruses (*Togaviridae*), modulate a series of similar HERVs at comparable levels, highlighting HERV4_4q12, HERVL40_9p21.1 and HERV30_11q14.1 (Figure 1B and Table 1). When analyzing the co-upregulation of HERVs between the four viruses, higher numbers of shared DEHs were shown in viruses belonging to the same family with a similar replication strategy (512 elements co-modulated by MayV and ChikV) (Figure 1C).

**Figure 2:**
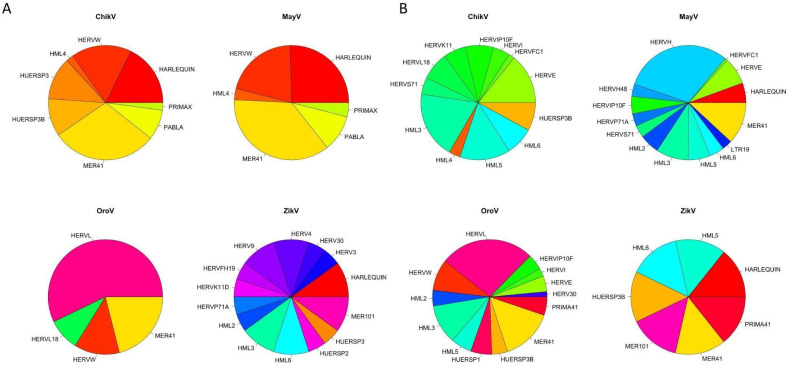
Enriched families of the differentially expressed HERVs by the arboviruses (ChikV, MayV, OroV, and ZikV). (**A**) Upregulated and (**B**) downregulated HERVs families.

Overall, except for ZikV, greater diversity was observed among HERVs families downregulated by arboviruses compared to the upregulated ones (Figure 2). Despite the low number of HERVs found, ZikV showed greater diversity in terms of upregulated element families. On the other hand, when observing the downregulated ones, ZikV presented itself as the least diverse, as would be expected due to the low number of elements found. OroV, in turn, showed a low diversity of upregulated HERV families, most of which belong to the HERV-L family. In contrast, the diversity of the downregulated ones was similar to that of alphaviruses.

The similarity between the ChikV and MayV results becomes even clearer when we look for the enriched HERV families, especially regarding the upregulated ones (Figure 2A). As for the downregulated HERV families, it can be observed that this equivalence between alphaviruses is somewhat lost, while the variety of modulated families increases (Figure 2B). The HERV families whose expressions were increased by MayV and ChikV viral infections were the same, and in very similar proportions, including HERVW, HARLEQUIN, PRIMAX, PABLA, MER41 and HML4. Only two families, HUERSP3 and HUERSP8, were upregulated by ChikV and not by MayV infection (Figure 2A). The present analysis reinforces a possible conserved role of HERVs upregulation within the alphavirus replication cycle.

### 3.2. HERV Upregulated Nearby Genes: Distance, Gene Ontology, Reactome and Integration Analysis

**Figure 3:**
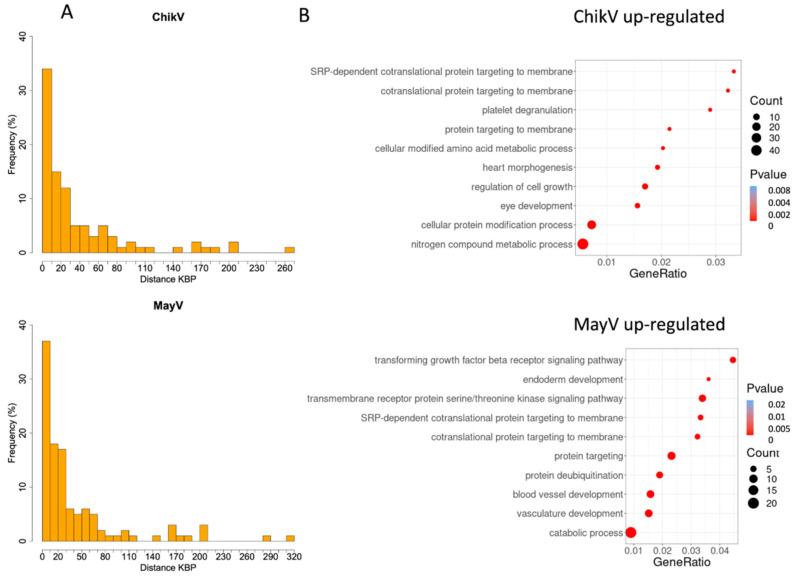
Genome positions and cellular pathways of nearby genes co-modulated by alphaviruses in human primary astrocytes cells. (**A**) Frequencies of the genes co-modulated by HERVs expressed through genomic position in kilobase pairs (KBP) in the alphavirus infections. (**B**) The most enriched GO categories of the nearest genes related to HERVs’ induction by alphaviruses.

It is known that the upregulation of HERV sequences also modulates the activity of nearby genes in a process called transcription interference [31]. As such, we search for the relationship between the distribution of HERV loci and these genes. The upregulated HERVs and their nearby genes, upstream and downstream, were correlated by comparing the HERVs’ and genes’ positions in the chromosome. Our results demonstrate that, for the four viruses, the majority of modulated HERVs are highly concentrated near the differentially expressed genes (Figure 3 and Figure S1).

Again, a similar pattern is observed between MayV and ChikV, reinforcing the possible evolutionary conservation of the gene expression control mechanism involving HERVs’ activation. For the differentially expressed gene loci, it is possible to see the upregulated HERVs inserted at up to 50 kb of their nearby genes, but the most are up to 10 kb (Figure 3A). However, it is interesting to also observe possible actions of HERVs in more distant genes, with a correlation of up to 260 kb for ChikV and 320 kb for MayV (Figure 3A).

To further explore the effects of Differentially Expressed Human Endogenous Retrotroviruses (DEHERVs) on gene regulation, we obtained transcriptome data referring to infection by the four agents in the same cell model in previously published work [24]. Due to the pattern of similarity between the alphaviruses observed so far, we found it interesting to explore the main categories of Gene Ontology (GO) represented among the upregulated DEGs close to the DEHERVs in the transcriptome data of these two viruses. Our results reinforce the role of HERV activation in regulating genes associated with the immune activation process. For example, among the main Gene Ontology (GO) categories of the HERVs’ nearby genes upregulated by MayV infection, there are genes related explicitly to the transforming growth factor beta (TGF-β) receptor signaling pathway, and among those upregulated by ChikV, related to platelet degranulation. In addition, pathways related to intracellular trafficking are also observed, which play an important role in cell-mediated signaling after viral infections.

**Figure 4:**
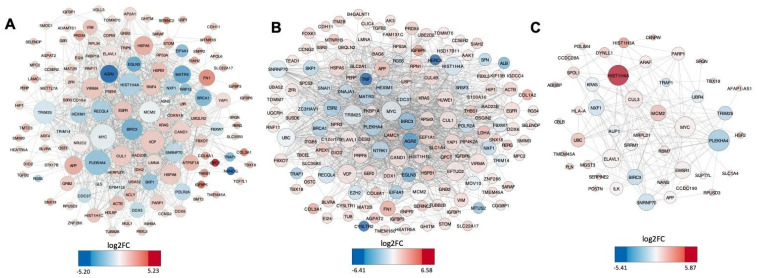
Networks of protein–protein interaction of DEGs related to the DEHERVs nearby genes. (**A**) ChikV; (**B**) MayV; (**C**) OroV.

Since the data referring to DEGs close to DEHERVs in the context of ZikV infection were not very robust at the time of infection analyzed (48 hpi), we decided to focus on a network analysis of the data of DEHERVs near to genes modulated by ChikV, MayV and OroV infections. Protein–protein (PIP) interaction networks were built with the DEG to detect possible signaling pathways (Table S1, Figure 4).

Each network is composed of a unique connected component with high connectivity. The interactome analysis of all DEGs shows action in the basic cellular function metabolism and regulation. This means most of the DEGs are hubs essential to cell functions. Again, the alphaviruses were more similar to each other, presenting 93 common genes. Among them, there were genes related to replication and cell cycle (*HIST1H1C*, *RERG* and *TMEM45A*), immune and antiviral response (such as *SARAF*, *ELAVL1*, *TGFB2*, *SERINC2*, *TRIM25*, *MOV10* and *TOMM70*), matrix and cytoskeleton elements (*COL3A1*, *COL8A1*, *FN1*, *ACTB*), ubiquitination pathways (*UBQLN2* and *USP1*) and other crucial and/or specific cellular pathways (including nervous system-related genes such as *MTNR1B*, *ENPP2*, and *APP*) (Table S1, Figure 4).

It was also possible to find 14 common DEGs among ChikV, MayV and OroV. This group includes a series of genes related to fundamental cellular pathways, such as *PLEKHA4*, *HIST1H4A*, *MCM2*, and *TMEM45A*, which have a role in cellular replication, and *NXF1* and *SNRNP70*, which are involved in gene transcription regulation. The antiviral and immune response was also represented by *KRAS*, *ELAVL1*, *TRIM25*, and *BIRC3* genes. Genes related to development, such as *TBX18* and *MYC*, were also found. The *APP* gene was also found in the networks and will be further discussed due to its role in Alzheimer’s disease. Taking these results into account, we gain insights into the families of HERVs and specific members that seem important in each of the infections, and between different infections, caused by arboviruses in astrocytes.

## 4. Discussion

Arboviruses are among the main infectious agents associated with the development of encephalitis. It is known that manifestations associated with these conditions can include seizures, memory impairment, confusion, and even leave neurological sequelae [21]. Recently, two cases of encephalitis associated with OROV were reported in northern Brazil [32], as well as another case of meningoencephalitis in the country’s southeastern region [33]. In addition, a triple neuronal infection caused by arboviruses (ChikV, OroV and Dengue type IV) was also recently identified in the state of Mato Grosso [34]. Such reports, especially when dealing with emerging viruses, are a serious public health issue that remains very much unknown.

It has already been described that viral infection alters the transcription pattern of HERV elements, mainly causing the induction of its expression. It is usually silenced in most somatic tissues due to epigenetic mechanisms [35,36,37,38]. In fact, the involvement of HERVs during the course of the viruses’ progression has been extensively studied, and they have even been found to promote viral replication and aggravate cases [39,40,41,42]. As an example, it was found that the expression of the ENV protein of the HERV-W family in the blood of patients positive for SARS-CoV 2 was correlated with inflammatory markers, in addition to being a predictor for the severity of the disease’s respiratory outcome [43]. Furthermore, the expression of endogenous retroviruses such as HERV-W and HERV-K18, at both mRNA and protein levels, has been explored during the development of chronic, inflammatory and autoimmune diseases [44,45]. It has been observed, for example, that the EBV protein LMP-2A is sufficient to transactivate a superantigen encoded by the HERV-K18 *env* gene, which is believed to be involved in the generation of autoreactive T lymphocytes [46]. The same transactivation was found during HHV-6B virus infection [47].

Recently, the first retrotranscriptional profile induced by dengue virus infection in A549 cells, named DENV2, was published [48]. Since DENV2 is an arbovirus, it is a very interesting profile for comparison with our data. In agreement with our findings, it was possible to observe both upregulated and downregulated HERVs, but the upregulated ones comprised the majority. However, similarities were also seen when looking at the families of HERVs that were downregulated between the arboviruses studied and DENV2 [48]. Among them, HML3 (downregulated in ChikV, MayV and OroV), HML4 (ChikV), HML5 (downregulated in all four arboviruses) and HML6 (ChikV, MayV and ZikV) stand out.

Most of the HERVs modulated in the studied viruses are located physically close to their neighboring genes. This finding agrees with what was seen for HERVs modulated by DENV-2 infection [48]. Genes correlated with upregulated HERVs found over greater distances were also observed. This is also reported in the literature, since it is already known that HERVs could act on genes spanning from several hundred kilobases to around one megabase [49,50]. The similarities found provide insights into which families of endogenous retroviruses may play a conserved role during the outcome of arbovirus infections.

The interactome analysis also showed us the possible influence of gene regulation caused by HERVs in basic processes involved in cell functioning. Histone cluster 1 H4 family member A (*HIST1H4A*), for example, is implicated in cell cycle progression and DNA replication processes, and was significantly downregulated in ZIKV-infected primary strains of human neural stem cells from gestational brains 9 and 13 weeks old [51]. Our data also demonstrate the downregulation of this specific histone in the alphaviruses interactomes, and upregulation in the OroV interactome. Additionally, the *MCM2* gene, active in repair during DNA replication and even used as a marker of an active cell cycle, showed a differential regulation between alphaviruses (decreased) and OroV (increased). These data evidence the resemblance among MayV and ChikV regulation patterns.

The nearby genes shared between the three viruses also provide evidence of the participation of HERVs as regulators of gene expression during the inflammation process generated by viral infection. In accordance with this antiviral state, our data show the upregulation of the *ELAVL1* gene in the reactomes of the three viruses. The *ELAVL1* gene transcript, also known as HuR, gives rise to an RNA-binding protein recognized as responsible for regulating the abundance of IFN-β mRNA, which regulates the type 1 IFN response [52].

Another interesting finding in our network is the differential modulation of transcripts noted as belonging to the *APP* gene, which encode the beta-amyloid precursor protein, known for its participation in Alzheimer’s pathogenesis. *APP* was upregulated in the infection by alphaviruses (ChikV and MayV) and downregulated in the OroV DEG data. The role of neuroinflammation generated by arboviruses in the central nervous system has been widely discussed, as well as its long-term consequences [21]. There is evidence that, for exogenous retroviruses such as HIV, CNS infection can lead to the development of brain disorders, such as amyotrophic lateral sclerosis or Alzheimer’s disease. The evidence further indicates that this phenomenon may involve the activation of HERVs [53,54]. Another interesting finding in this context is the self-renewal gene *NANOG*, which is downregulated in the CHIKV interactome. *NANOG* is part of the group of factors involved in the fine spatio-temporal regulation that coordinates embryonic neurodevelopment, along with other genes such as *HOXA1*, *SOX2* and *MYC* [55]. Therefore, these predictions open the door to questions and possible studies regarding the role of alphaviruses (and their associated HERVs) in developing future neurological conditions.

Despite the apparent differences cited between the regulatory patterns observed for OroV and alphaviruses, some genes remained regulated in the same direction. *TRIM25* stands out here. This gene encodes for an antiviral protein and was downregulated in the three reactomes. TRIM25 binds to viral RNA genomes and promotes the recognition of viral replication, inducing premature uncoating and capsid disassembly [56,57]. Likewise, *TRAF1*, the gene involved in the recognition of TNF, was also downregulated in the three reactomes. Observing our data, we hypothesized that HERVs regulation may be involved in modulating some steps of the cellular defense system against arboviral infections.

As mentioned before, one consistent finding among our data was the high similarity in the HERVs modulated by ChikV and MayV. This similarity was presented both in relation to the diversity of families, as well as in the abundance and identity of the isolated HERVs. In this context, the HERV-W family is an interesting finding, as it is already associated with the pathogenesis of nervous system disorders, such as multiple sclerosis, corroborating the possible role of these elements in the development of neuroinflammation [58].

Like other viral agents, alphaviruses have evolved mechanisms to promote their replication and deal with the antiviral response to reduce the cell’s antiviral status and prevent warning signals from being distributed to uninfected cells. The PPI interactomes reinforce the participation of HERVs as possible agents of gene regulation acting during the processes resulting from the infection, promoting an antiviral and pro-inflammatory environment in these agents. Examples of this process may include the predictions found for regulating genes such as *MOV10*, *BIRC3*, *SERINC2* and others previously mentioned.

It is well established that alphavirus infections are sensitive to type I IFN [59]. A nearby gene relevant in this context is *DDX6*. This DEAD-box helicase, present in *p*-bodies and stress granules, had its expression slightly increased in alphaviruses reactomes. For DENV2, a relevant role of DDX6 is observed in promoting the viral replicative cycle. Ward et al. (2011) demonstrated that *DDX6* knockdown resulted in reduced amounts of infectious viral particles and DENV-2 viral RNA in supernatants derived from infected HuH-7 cells. The same work also demonstrated an interaction with the elements of secondary structures DB1 and DB2, present in the 3 ‘UTR region of the viral genome, thus being a fundamental step in viral replication [60]. The promoting effect of the *DDX6* in viral replicative cycles has also been proven for several other viruses (such as HCV, HIV and FV) in several stages such as translation, genome replication and capsid assembly [61]. However, the increase in the DDX6 protein promotes an antiviral state in the cell since it interacts with RIG-1 (intracellular viral RNA sensing molecule) in the stress granules, promoting the expression of the β-type interferon. It is also known that the interferon alpha/beta (IFN-α/β) response is a first-line innate defense against arboviruses, including ChikV [62]. In addition, DDX6 can also act as a co-sensor for RIG-1 since the molecule binds to the viral RNA capable of stimulating RIG-1 [63].

We can also see a molecular picture that appears to be the result of the antiviral response in astrocytes. For example, the expressions of two collagen forms (from *COL3A1* and *COL8A1* genes) were slightly increased in both alphaviruses. In particular, *COL8A1* seems to play an important role in the reactomes. These data differ from what is seen for ZIKV infection in children with Congenital Syndrome, where the specific expression of *COL8A1* is reduced [64]. This finding starts to make sense since it is known that astrocytes express type VIII collagens to repair brain tissue injuries [65]. It is also known that the production of these specific collagens in astrocytes and other cell types is related to the TGF-β2 expression [66,67], which is also slightly upregulated in our alphavirus reactomes.

Molecules related to the stimulation of viral replication were also found in the reactomes. The *OSTC* gene, whose product participates in the production of asparagine during N-glycosylation, was also increased in ChikV and MayV reactomes. The OST complex was found to be fundamental to DENV; for example, DENV replication was nearly absent in cells deficient for this complex [68]. Among the HERV’s nearby genes differentially expressed in the alphavirus data, the category “SRP-dependent cotranslational protein target to membrane” was observed. The SRP protein is part of the cell trafficking system. Its function is to interact with signal peptides present in nascent proteins so as to forward them to the plasma membrane for integration or secretion. A study analyzing the host–parasite interaction concerning this pathway in SARS-CoV-2 infection concluded that the viral proteins NSP7 and NSP9 interact with SRP to disrupt the secretion of host proteins in the context of decreasing the antiviral response via IFN [69]. This category was also found in the GO referent to differentially expressed genes modulated by HERVs in DENV2 infection [48], probably due to the migration of viral surface proteins to the plasma membrane, as has already been pointed out for influenza [70]. Together, such findings may suggest that the upregulation of HERVs could play an important role in the regulation of gene transcription that results in the modulation of the inflammatory response triggered by a viral infection and promotes viral replication and spread.

## 5. Conclusions

Our data show a pattern in the regulation of HERVs and nearby genes common in arboviruses that cause encephalitis, but greater similarity was shown between viruses of the same family, as seen for alphaviruses, with most HERVs being upregulated. Through this first prospective analysis, it is possible to start thinking about studies that aim to further explore these data, aiming to use these endogenous retroviruses as biomarkers in terms of the diagnosis and progression status of inflammatory conditions caused by those agents.

## Figures and Tables

**Table 1:** Differentially expressed HERVs present in all viruses.

HERV	ChikV		MayV		OroV		ZikV	
	log2FC	q-Value	log2FC	q-Value	log2FC	q-Value	log2FC	q-Value
ERV316A3_12q24.13	6.366	1.90 × 10^−6^	7.004	4.73 × 10^−8^	8.006	3.94 × 10^−9^	5.681	9.04 × 10^−5^
ERV316A3_3q27.3e	3.946	0.00099	5.164	1.22 × 10^−6^	3.913	0.001330	3.228	0.021470
ERV316A3_7q34a	6.362	1.29 × 10^−5^	7.813	8.46 × 10^−9^	6.249	0.000368	3.913	0.048601
ERVLB4_12p13.2a	7.738	9.68 × 10^−10^	8.414	9.95 × 10^−12^	5.440	2.98 × 10^−5^	5.266	0.000273
HARLEQUIN_17q12	3.698	9.65 × 10^−7^	3.860	4.38 × 10^−8^	6.680	3.17 × 10^−6^	3.946	7.72 × 10^−8^
HERV4_4q22.1	4.520	4.21 × 10^−25^	5.181	4.01 × 10^−36^	5.665	6.00 × 10^−53^	4.734	7.63 × 10^−30^
HERV9_11q21	3.363	1.01 × 10^−11^	3.886	8.34 × 10^−18^	2.613	7.21 × 10^−5^	1.773	0.003343
HERVK11D_2q11.2	4.270	4.14 × 10^−10^	4.494	3.63 × 10^−12^	2.058	0.035437	2.051	0.025747
HML3_12q13.12	7.832	9.26 × 10^−9^	8.662	5.28 × 10^−11^	6.631	3.77 × 10^−5^	5.694	0.000241
HML3_16p13.3	7.785	1.21 × 10^−8^	8.547	1.01 × 10^−10^	6.079	0.001019	3.909	0.046599
HML6_14q24.2	9.465	4.98 × 10^−13^	9.569	1.42 × 10^−13^	9.166	3.57 × 10^−12^	5.300	0.000859
HML6_19p13.2c	5.274	3.84 × 10^−7^	5.421	6.02 × 10^−8^	2.418	0.000147	3.131	0.018769
HUERSP3_2p25.2	7.397	6.92 × 10^−8^	8.553	7.31 × 10^−11^	9.796	2.60 × 10^−13^	7.404	1.56 × 10^−7^
MER101_1p22.2a	3.510	2.21 × 10^−8^	3.662	3.83 × 10^−10^	3.876	3.34 × 10^−11^	2.064	0.007506
MER101_2p25.2	6.205	6.93 × 10^−8^	7.081	1.47 × 10^−10^	4.254	0.013413	6.832	6.34 × 10^−9^

## Data Availability

Data are available via a web application at https://biotools.labinfo.lncc.br/hervs_astrocytesvirus, accessed on 14 September 2022. The results are organized in a dynamic search database with enrichment analysis using Gene Ontology, KEGG pathway and Reactome, with Venn diagram comparisons between the two contrasts.

## Supplementary Materials

The following supporting information can be downloaded at: https://www.mdpi.com/article/10.3390/v14112505/s1, Table S1: Differentially expressed HERVs with the nearby gene expression of ChikV, MayV, OroV and ZikV; Figure S1: Frequencies of the genes co-modulated by HERVs expressed through genomic position in kilobase pairs (KBP) in the OroV and ZikV infections.
