# Supplementary figures and images for: Modulation of HERV Expression by Four Different Encephalitic Arboviruses during Infection of Human Primary Astrocytes
Source: Viruses. 2022 Nov 12;14(11):2505. doi: 10.3390/v14112505 (PMC9694637; doi:10.3390/v14112505)

**A****OroV**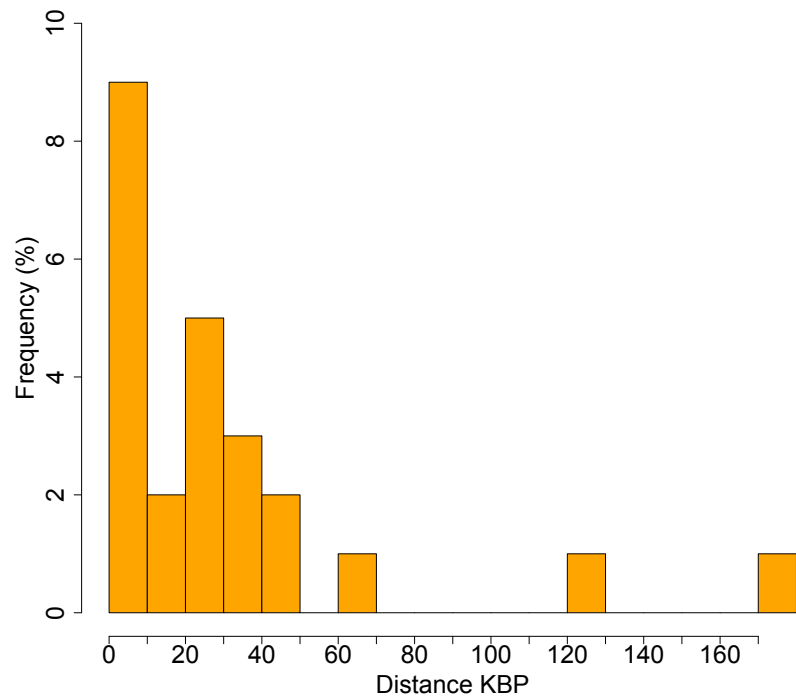**B****ZikV**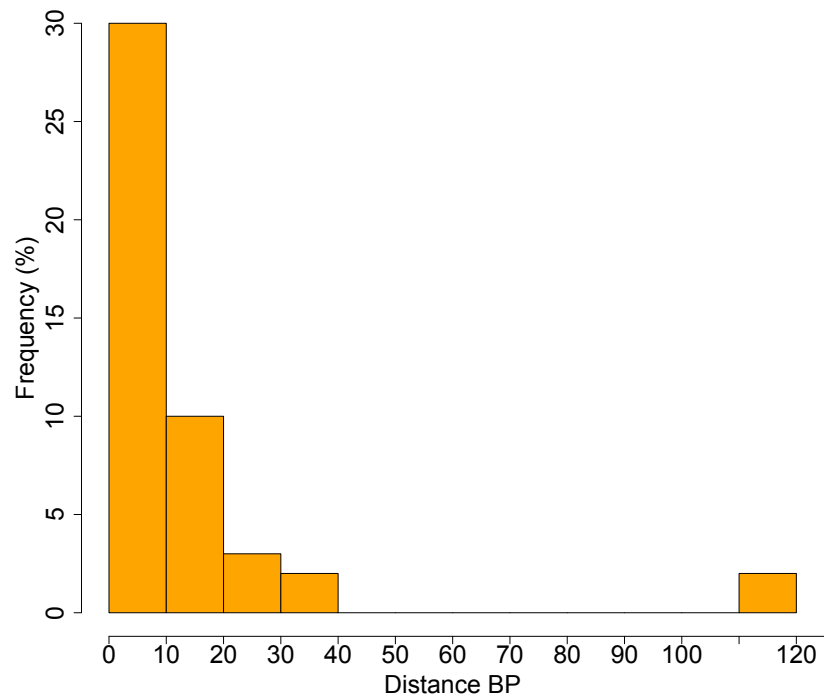

Supplement: Supplementary file 1 [file viruses-14-02505-s001.zip › Figure S1.pdf]
